# The interactions and essential effects of intrinsic insulin-like growth factor-I on *Leishmania (Leishmania) major* growth within macrophages

**DOI:** 10.1111/pim.12041

**Published:** 2013-07-01

**Authors:** L C Reis, E M Ramos-Sanchez, H Goto

**Affiliations:** 1 Instituto de Medicina Tropical de São Paulo, Universidade de São Paulo, IMTSP-USPSão Paulo, Brazil; 2 Departamento de Medicina Preventiva, Faculdade de Medicina, Universidade de São PauloSão Paulo, Brazil

**Keywords:** confocal microscopy, insulin-like growth factor-I, *Leishmania (Leishmania) major*, macrophage, Interferon-γ

## Abstract

Previously, we showed in *Leishmania* infections that extrinsic insulin-like growth factor (IGF)-I favored *Leishmania* proliferation and leishmaniasis development. In this study, the interaction of intrinsically expressed IGF-I and *Leishmania* (*Leishmania*) *major* in macrophages was addressed, and a key finding was the observation, using confocal microscopy, of the co-localization of IGF-I and parasites within macrophages. Following stimulation with interferon-γ (IFN-γ), which is known to inhibit IGF-I production in macrophages, we observed a reduction in the expression of both IGF-I mRNA and protein. This reduced expression was accompanied by a reduction in the cellular parasite load that was completely recovered with the addition of extrinsic IGF-I, which suggests an essential role for IGF-I in *Leishmania* growth.

Leishmaniases are diseases that are caused by heteroxenic protozoans, which belong to the genus *Leishmania* (Kinetoplastida: Trypanosomatidae). Leishmaniases are transmitted vectorially and affect more than 12 million people in tropical and subtropical areas of the world. The lesions present in infected patients may affect the skin, mucosa, cartilage or viscera 1.

In leishmaniasis, the parasite–host interaction begins immediately after the inoculation of the parasite into the host, where many growth factors exert their effects as nonspecific factors or as supporting elements in the adaptive immune response that ultimately controls parasite proliferation 2. Among the growth factors, we focused on the insulin-like growth factor (IGF)-I. IGFs are phylogenetically well-preserved polypeptides with a molecular mass of approximately 7·5 kDa. We have studied two known major forms, IGF-I and IGF-II, in *Leishmania* infection 3. We initially showed that the addition of physiological concentrations of extrinsic IGF-I into cultures induced the increased proliferation of different species of *Leishmania* promastigotes and axenic amastigotes, an effect that was not seen with IGF-II despite its great similarity to IGF-I 4–6. In experimental models, extrinsic IGF-I induced significant increases in lesion sizes and in the number of viable parasites at the lesion sites 7. *In vitro*, IGF-I favored parasite growth in *Leishmania* (*L*.) *amazonensis*-infected macrophages through an increase in arginase expression and activity in both parasites and macrophages, as well as through a decrease in the production of nitric oxide by macrophages 8.

However, host macrophages that harbour amastigotes express intrinsic IGF-I 9; thus, we asked whether intrinsic IGF-I effects and interacts directly with *Leishmania* or, alternatively, if it acts through the activation of another intermediary factor within macrophages. In this work, we addressed the interaction of macrophage intrinsic IGF-I with *Leishmania (Leishmania) major*. We evaluated the localization of IGF-I and its association with the parasite using confocal microscopy and analysed whether modulation of IGF-I expression might affect intracellular parasite growth. Knowing that Th1 cytokines inhibit IGF-I expression in macrophages 10, we used interferon-gamma (IFN-γ) as a stimulus to reduce IGF-I expression and to evaluate the consequential effects on parasitism in *L. major*–infected macrophages. For these experiments, RAW 264·7 cells (macrophage cell line, ATCC) were grown in DMEM medium (Sigma, St. Louis, MO, USA) that was supplemented with 0·5% bovine serum albumin (BSA; Sigma). The cells were dispensed (5 × 10^5^ or 2 × 10^6^) onto round 13 mm^2^ glass cover slips that were placed in the wells of 24-well plates (Corning Costar, Corning, NY, USA) and allowed to adhere for 30 min at 37°C in a humid atmosphere with 5% CO_2_, followed by two washes with culture medium to remove nonadherent cells. Next, a *L. major* LV 39 (MRHO/Sv/59/P) promastigote suspension (at a ratio of 8 parasites per cell) was dispensed into the wells and allowed to infect the cells for 4 h at 33°C in a humid atmosphere with 5% CO_2_, followed by a wash step to remove the noninternalized parasites. In some experiments, the macrophages were treated with IFN-γ (200 U/mL; BD Biosciences, San Jose, CA, USA), and the cultures were maintained for 48 h. In some experiments, recombinant IGF-I (50 ng/mL; rIGF-I, R&D Systems, Minneapolis, MN, USA) was added. In a set of experiments for analysis, the expression and localization of IGF-I and *Leishmania* within the macrophages were analysed using confocal microscopy and immunofluorescence staining. Following a 24-h *in vitro* infection, cells were fixed in 4% paraformaldehyde (Sigma), washed in 0·001 m phosphate buffered saline, pH 7·2 (PBS), blocked for 1 h with 2% BSA in PBS, and incubated overnight with monoclonal goat anti-mouse IGF-I antibody (1 : 75; R&D Systems) and a polyclonal mouse anti-*Leishmania* antibody (1 : 400), which was produced in our laboratory 11. Anti-goat IgG Alexa Fluor-546 (1 : 200, Invitrogen, Carlsbad, CA, USA) and anti-mouse IgG Alexa Fluor-488 (1 : 400, Invitrogen) were used as secondary antibodies. Fluorescence image studies were performed using a Zeiss LSM 510 META laser-scanning confocal microscope (Carl Zeiss, Oberkochen, Germany). In negative controls, the primary antibodies were omitted from the reactions. All experimental procedures were approved by the ethics committee of the institution.

In the initial approach to evaluate macrophage intrinsic IGF-I and *Leishmania* using confocal microscopy, IGF-I was seen to be distributed in all cytoplasmic regions and around *Leishmania* (Figure 1a,b), which revealed a direct interaction between the parasite and IGF-I. Cytoplasmic IGF-I expression is not unique to macrophages, as it has also been observed in the cytoplasmic regions of other type of cells, such as neurons, chondrocytes, pancreatic islet cells and mesenchymal stem cells 12–15. We also observed similar cytoplasmic expression in mouse peritoneal macrophages (data not shown). This co-localization of IGF-I and *Leishmania* within macrophages was shown here for the first time. Importantly, in cultured free promastigotes in the absence of macrophages, IGF-I staining was absent (Figure 1c). This result was somewhat expected, because *Leishmania* does not express IGF-I. However, because the parasite expresses the receptor for IGF-I 16 once it is internalized within macrophages, it likely responds to the IGF-I that is produced by these cells.

**Figure 1:**
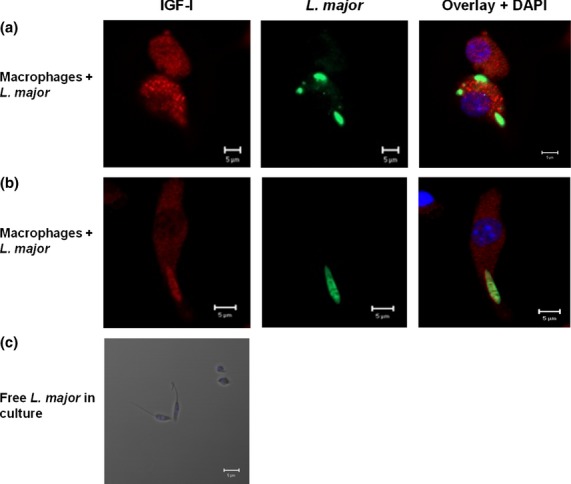
Detection of IGF-I within RAW 264·7 macrophages following infection with *Leishmania major* promastigotes. Co-localization of IGF-I and *Leishmania* was measured using immunofluorescence. Anti-IGF-I antibody (recognized by the secondary antibody AlexaFluor-546, shown in red) and anti-*Leishmania* antibody (recognized by the antibody AlexaFluor-488, shown in green) were used to label cells (a, b). Free *L. major* promastigote culture immunofluorescence was measured using anti-IGF-I and the secondary antibody AlexaFluor-546 (c). No IGF-I staining was observed (red). 4′,6-diamidino-2-phenylindole (DAPI, shown in blue) was used to stain nuclei. Images were captured with a Leica LSM510 confocal microscope with a 63× objective and oil immersion.

Having demonstrated the co-localization of IGF-I and parasites in the infected macrophages, we initially intended to evaluate the role of intrinsic IGF-I in *Leishmania* infections. For this purpose, we used IFN-γ to stimulate the cells, as it is known to have an inhibitory effect on IGF-I expression 10, enabling an analysis of the effect of IGF-I on parasitism. We initially analysed the effects of IFN-γ on the expression of IGF-I messenger RNA (mRNA) in *L. major*–infected and noninfected control macrophages. To evaluate this expression, total RNA was extracted from 2 × 10^6^ cells/mL using TRIzol (Invitrogen), following the manufacturer's protocol (RNA integrity was determined as an OD260/280 absorption ratio >1·8). Next, 1 μg of purified RNA was mixed with 12 μL of a solution consisting of a basic buffer (100 mm Tris-HCl, pH 8·3, containing 500 mm KCl and 15 mm MgCl_2_ Invitrogen), dNTP (10 mm; Fermentas, Vilnius, Lithuania), random primers (Invitrogen), OligoDT primers (Invitrogen), RNaseOUT recombinant ribonuclease inhibitor (40 U/μL; Invitrogen), M-MLV reverse transcriptase (100 U/μL). The reactions were incubated at 37°C for 50 min and were denatured at 70°C for 15 min. For real-time quantitative RT-PCR, the following primer set for murine IGF-I was designed: forward, 5′ TAC TTC AAC AAG CCC ACA GG 3′ and reverse, 5′AGT CTT GGG CAT GTC AGT GT 3′ (GenBank accession no. NM010512). β-actin (GenBank accession no. NM00739) was used as a constitutively expressed control gene for normalization (primers: forward, 5′ GCC TTC CTT CTT GGG TAT GGA ATC 3′ and reverse, 5′ ACG GAT GTC AAC GTC ACA CTT CAT 3′). The reactions included master mix (SYBR®Green; Applied Biosystems, Foster City, CA, USA) and 1 μL cDNA template and were run in triplicate on a PCR system (StepOne; Applied Biosystems). The PCR conditions were the same for all primer combinations: 95°C for 10 min, 40 cycles of 92°C for 2 min, 57·5°C for 30 s and 70°C for 30 s. After PCR amplification, a melting curve was generated to confirm the specificity of the product. The data were presented as a relative quantification and were calculated using 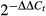 17.

As expected, IGF-I mRNA was expressed in uninfected control macrophages and IGF-I mRNA expression was decreased when the cells were infected (Figure 2c). This slight decrease in the IGF-I mRNA expression with promastigote infection could be an evasion mechanism of the parasite. It is known that the parasite has glycoproteins and sugars in the membrane that exert important roles in the modulation of cell signalling in the host that may participate in the modulation of IGF-I expression. The glycoprotein 63 (metalloproteinase gp63), the most abundant glycoprotein on the parasite surface, may be involved in this decrease in the expression because it has been shown that this glycoprotein from different species of *Leishmania* (*L. donovani, L. mexicana, L. major*) inhibits the activity of an important transcription factor, AP-1, in macrophages that are important to increase the IGF-I expression 18, 19. The gp63 can inhibit the activity of AP-1 resulting in no induction of IGF-I transcription.

When infected cells were stimulated with IFN-γ, we observed a 6·9-fold decrease in IGF-I mRNA expression (Figure 2c). This decrease in IGF-I mRNA expression was also seen concomitantly at the qualitative protein level, using confocal microscopy, with which we observed a decrease in IGF-I immunostaining in the IFN-γ-stimulated cells (Figure 2d,e). These results corroborate what has been reported in the literature on the effects of cytokines on IGF-I expression, particularly decreased expression following IFN-γ stimulus 10, 20.

**Figure 2:**
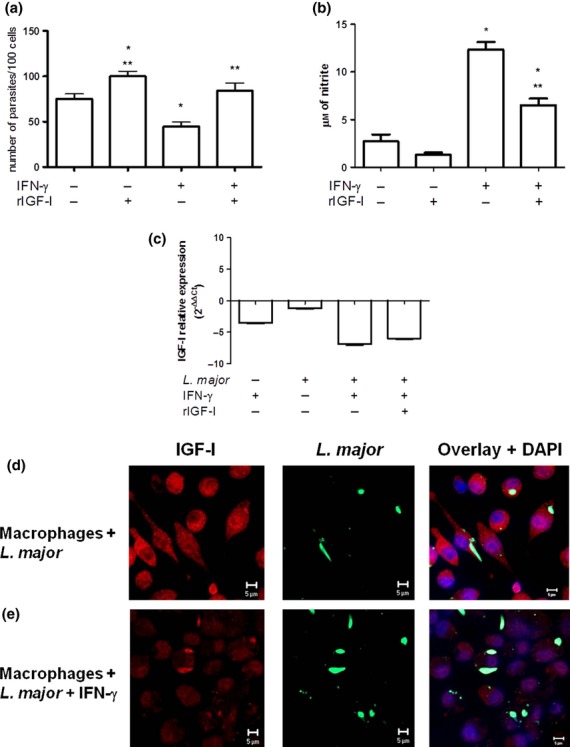
Parasitism, nitric oxide (NO) production, IGF-I mRNA and IGF-I protein expression in RAW 264·7 cells that were infected with *Leishmania major* promastigotes. (a) Parasitism (shown as the number of parasites per 100 cells), (b) NO as nitrite levels evaluated in culture supernatants and (c) IGF-I mRNA expression (calculated as the ratio in relation to the control group without stimulation, 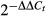) with and without IFN-γ (200 U/mL) stimulus and with recombinant IGF-I (rIGF-I, 50 ng/mL) stimulus during a 48 h incubation. The data shown are representative of three independent assays. (d, e) Detection of IGF-I by confocal microscopy, using anti-IGF-I antibody (recognized by the secondary antibody AlexaFluor-546, shown in red) and anti-*Leishmania* antibody (recognized by the secondary antibody AlexaFluor-488, shown in green). 4′,6-diamidino-2-phenylindole (DAPI, shown in blue) was used to stain the nuclei. Images were captured using a confocal Leica LSM510 confocal microscope with a 63× objective and oil immersion. *P < 0·05 (anova and Tukey's test) in relation to the control group. **P < 0·05 (anova and Tukey's test) in relation to the IFN-γ group.

To examine the effects of decreased IGF-I expression on parasitism, macrophages (5 × 10^5^ cells) were infected with *L. major* promastigotes and stimulated with IFN-γ. Parasitism was evaluated under light microscopy (Carl Zeiss), and 600 cells were counted per group. The data were presented as the number of parasites per 100 cells [(number of parasites/number of infected cell) × (number of infected cells/total number of cells) × 100]. Nitric oxide (NO) production was evaluated by the nitrite (NO_2_) accumulation in the supernatants of cell culture as an indicator of NO production and was determined by a standard Griess reaction 21. Fifty microlitres of the culture supernatant was reacted with 50 μL of Griess reagent (1% sulphanilamide, 0·1% naphthylethylene diamine dihydrochloride, 2·5% phosphoric acid in bidistilled water) for 10 min at room temperature. The absorbance was measured at 540 nm using a Multiskan MCC/340 P version 2.20 plate reader (Labsystems, Vienna, VA, USA), and the nitrite concentration was calculated using a standard curve of sodium nitrite (NaNO_2_). Data were submitted to statistical analysis by anova and Tukey's tests and were considered significant when *P* < 0·05.

Analysing the parasite load in the control cells without stimulus, we observed 75 parasites per 100 cells (median). Upon IFN-γ-stimulus, we observed a significant decrease in the parasite number to 45 per 100 cells (*P* < 0·05) (Figure 2a). This decrease in parasite load was accompanied by a reduction in IGF-I mRNA expression and an increase in NO production by cells after IFN-γ stimulus (*P* < 0·05) (Figure 2b).

To ascertain the role of IGF-I on parasitism, we reconstituted the culture with extrinsic IGF-I. In the control group, the addition of recombinant IGF-I (rIGF-I – 50 ng/mL) led to an increase in the number of parasites to 98 per 100 cells when compared with controls (*P* < 0·05). In the IFN-γ-stimulated culture, the addition of rIGF-I also led to an increase in the number of parasites to 84, a level similar to the parasite load that was seen in controls with or without rIGF-I (Figure 2a) and with no increase in the IGF-I mRNA expression (Figure 2c). In cells stimulated with IFN-γ and rIGF-I, we observed a decrease in the NO production when compared with cells stimulated with IFN-γ but still higher than in control without IFN-γ-stimulus (Figure 2b). Therefore, the effects observed in parasitism were likely due to the added extrinsic IGF-I.

The ability of IGF-I to circumvent the effect of IFN-γ may be explained by our previous finding which showed a role for IGF-I in the induction of the alternative activation of macrophages 8. Macrophage activation leads to the generation of different metabolic products of L-arginine. When L-arginine is catalysed by inducible nitric oxide synthase (iNOS), nitric oxide (NO), a leishmanicidal product that is indispensable for the control of the parasite, is produced. However, when L-arginine is hydrolysed by arginase, polyamines are generated that are essential nutrients for *Leishmania* growth 22, 23. The present results suggest that macrophage intrinsic IGF-I also affects this pathway. Further, this hypothesis is supported by a study in which the inhibition of the intracellular killing of parasites in IFN-γ-treated cells was observed when arginase was added to the culture media 24. Overall, our results suggest an essential role for IGF-I in *Leishmania* growth within macrophages and apparently circumventing the effect of IFN-γ.

In this study, we report the co-localization of intrinsic IGF-I and *Leishmania* and suggest a direct interaction and effect of IGF-I on *Leishmania* within infected macrophages. Further, we show a parallel reduction in both IGF-I expression and parasite load following IFN-γ stimulus, and a recovery of the parasite load following the addition of extrinsic IGF-I, which suggests the crucial involvement of intrinsic IGF-I in *Leishmania* parasite growth within macrophages. This study opens a broad area of research on the interactions of IGF-I and Th1 and Th2 cytokines with related signalling pathways.
